# Azole Fungicides and Their Endocrine Disrupting Properties: Perspectives on Sex Hormone-Dependent Reproductive Development

**DOI:** 10.3389/ftox.2022.883254

**Published:** 2022-04-28

**Authors:** Monica Kam Draskau, Terje Svingen

**Affiliations:** National Food Institute, Technical University of Denmark, Lyngby, Denmark

**Keywords:** fluconazole, reproduction, conazoles, endocrine disrupting chemicals, clotrimazole, reproductive disorders, testis, androgen

## Abstract

Azoles are antifungal agents used in both agriculture and medicine. They typically target the CYP51 enzyme in fungi and, by so doing, disrupt cell membrane integrity. However, azoles can also target various CYP enzymes in mammals, including humans, which can disrupt hormone synthesis and signaling. For instance, several azoles can inhibit enzymes of the steroidogenic pathway and disrupt steroid hormone biosynthesis. This is of particular concern during pregnancy, since sex hormones are integral to reproductive development. In other words, exposure to azole fungicides during fetal life can potentially lead to reproductive disease in the offspring. In addition, some azoles can act as androgen receptor antagonists, which can further add to the disrupting potential following exposure. When used as pharmaceuticals, systemic concentrations of the azole compounds can become significant as combatting fungal infections can be very challenging and require prolonged exposure to high doses. Although most medicinal azoles are tightly regulated and used as prescription drugs after consultations with medical professionals, some are sold as over-the-counter drugs. In this review, we discuss various azole fungicides known to disrupt steroid sex hormone biosynthesis or action with a focus on what potential consequences exposure during pregnancy can have on the life-long reproductive health of the offspring.

## Introduction

Over-the-counter (OTC) drugs are widely accessible. The global pharmaceutical OTC drugs market is estimated to be around US $130 billion per annum and steadily increasing. These drugs are deemed safe for the consumer as long as they are used appropriately. However, used inappropriately even OTC drugs pose potential health risks and should be used with caution. Worryingly, a high proportion of consumers consider OTC drugs completely harmless, thereby running the risk of adverse health effects by underestimating the risks associated with self-medication (Roumie and Griffin, 2004; Westerlund et al., 2017; Ylä-Rautio et al., 2020; Bauer et al., 2021). For instance, a Swedish survey from 2017 reported that 7% of participants agreed completely or to a large extent on the statement that OTC drugs are “*completely harmless regardless of how they are being used*” and 54% that they are *“harmless as long as you follow the directions on the package”* (Westerlund et al., 2017)*.*

OTC drugs comprise a diverse group of pharmaceuticals counting more than 300,000 registered products according to the United States Food and Drug Administration (U.S. FDA, 2020). They have widely different applications and effects, also with varied drug-related problems (Ylä-Rautio et al., 2020). Amongst these effects are endocrine disrupting properties and consequences for reproductive development and function. In particular mild analgesics have received much focus in recent years because of suspected endocrine disrupting properties and associated reproductive disorders (Kristensen et al., 2016; Bauer et al., 2021). A major concern with such endocrine disrupting OTC drugs is inappropriate use by pregnant women, as fetal sexual development is reliant on sex hormones. If these hormones are disrupted during early life, especially in male fetuses, it can lead to a range of reproductive disorders in humans including genital malformations, germ cell cancers, and infertility (Skakkebaek et al., 2016; Jorgensen et al., 2021).

Besides analgesics, OTC antifungal drugs containing azoles are widely used, also by pregnant women who frequently develop vaginal yeast infections (Sobel, 2007). Many azole fungicides are potent endocrine disruptors, but these also include both prescription drugs and agricultural pesticides. With regard to OTC drugs containing azoles as the active ingredient, they are considered safe for the user; however, recent studies have questioned this, at least for certain vulnerable individuals such as the unborn child (Mogensen et al., 2017; Draskau et al., 2021). Herein, we will briefly discuss the endocrine disrupting potential of azoles and possible detrimental effects on sexual development.

## Azole Fungicides

The first azole with antifungal activity was reported in the 1940s and by the late 1950s antifungal drugs with azoles as the active ingredient was introduced to the market for clinical use (Sheehan et al., 1999). Today, more than 60 years later, the number of azole compounds has increased and they are used extensively to combat yeast infections in a clinical setting; often a first-choice treatment for mycotic infections including vaginal mycosis in pregnant women (Sobel, 2007). Notably, azoles are also widely used for agricultural crop protection. These agricultural azoles are not a focus of this review, but rather mentioned for two important reasons. Firstly, because the broad use in both agriculture and pharmacology leads to emerging fungal resistance to azoles (Bastos et al., 2021). Secondly, because the endocrine disrupting modes of action often are shared between many agricultural and medicinal azoles, thus studies across domains can inform on their potential to cause endocrine disrupting effects.

Azole antifungal agents, being it agricultural or medicinal, are classically designed to interfere with the heme-group of the fungal cytochrome P450 (CYP) enzyme CYP51, also known as sterol 14α-demethylase (Podust et al., 2001; De Petris et al., 2015). In this way, azoles block the production of the essential fungal membrane component ergosterol, disrupting the cell membrane and ultimately inhibiting fungal growth (Hitchcock et al., 1990; Georgopapadakou, 1998). A challenge, however, is that CYP enzymes are found in all animal kingdoms. For instance, humans and other animals also possess a CYP51 enzyme that is structurally closely related to fungal CYP51. But perhaps more problematically, many azoles can also interfere with other members of the CYP superfamily of enzymes, of which there are many.

With respect to reproductive development, which is the primary focus herein, the CYP enzymes of the steroidogenic pathway are obvious and include important regulators of sex hormone synthesis such as CYP17 and CYP19. In fact, CYP19 (aromatase) has been shown to be a key target of many azole fungicides, both agricultural and medicinal (Mason et al., 1985; Vinggaard et al., 2000; Kragie et al., 2002; Zarn et al., 2003; Trösken et al., 2006). In humans and other mammals, CYP19 is responsible for converting androgens to estrogens and as such play a critical role in sex hormone homeostasis throughout development and in adulthood (Simpson et al., 2002; Blakemore and Naftolin, 2016). Hence, perturbing CYP19 activity can impede on sexual development and function, as discussed further below.

## Hormone-Dependent Male Sexual Development

A detailed description of male sexual development is beyond the scope of this article, yet we will outline some key points that are essential with respect to the potential adverse effects azoles can cause if exposure occurs during fetal life. In mammals, sex is initially determined by the pairing of sex chromosomes at fertilization, which is followed by gonadal sex determination approximately 6 weeks after conception in humans (Jorgensen et al., 2021), resulting in testes forming in XY fetuses and ovaries in XX fetuses. Following gonadal sex determination, the testes of male fetuses will rapidly differentiate and start to produce testosterone by the fetal Leydig cells (Svingen and Koopman, 2013). In turn, testosterone will be secreted by the testes into the circulation and, in simple terms, drive masculinization of the male fetus. In rats, a masculinization programming window (MPW) has been established (Welsh et al., 2008), which corresponds to a window of fetal development where most of the masculine phenotypes are determined in response to a surge in testosterone synthesis; a sensitive window also found in other mammals, including humans (Sharpe, 2020). Importantly, however, differentiation of external genitalia and several other androgen-sensitive tissues relies on the presence of dihydrotestosterone (DHT) rather than testosterone.

DHT is synthesized from testosterone by the enzyme 5α-reductase in target tissues. In mice and rats, this relationship appears relatively linear, with testosterone mainly deriving from the testicular Leydig cells. However, in several other mammals, including humans, DHT can also be synthesized from the intermediate steroid hormone androsterone during development and this intermediate steroid is largely derived from the placenta, and to a lesser extent the fetal liver and adrenals (O’Shaughnessy et al., 2019). This alternative ‘backdoor pathway’ to androgen signaling during development is significantly different between species such as rats and humans, and must be considered when evaluating how perturbation to androgen synthesis or signaling can result in incomplete masculinization (Sharpe, 2020).

Nevertheless, exposure to chemical substances with anti-androgenic activities during the MPW can impede on masculinization processes and lead to reproductive disorders. They include hypospadias or cryptorchidism at birth, or reduced fertility or testis cancers later in life (Skakkebaek et al., 2016; Jorgensen et al., 2021). Impaired masculinization can also be assessed by the anogenital distance (AGD), which is considered a broad biomarker of fetal androgen action; although not a disorder in itself, a short AGD in male offspring is associated with male reproductive disorders (Thankamony et al., 2016; Schwartz et al., 2019; Fischer et al., 2020). As will be discussed in the following, exposure to certain azoles can lead to these adverse effect outcomes.

## Adverse Reproductive Effects of Azole Fungicides

Prenatal exposure to antifungal azole drugs has been associated with short male AGD in humans (Mogensen et al., 2017). This preliminary study reported that exposure to a single oral dose of 150 mg fluconazole inside gestational weeks 8–14 of pregnancy (assumed to be the human MPW) was associated with a significantly shorter AGD in newborn boys. Interestingly, use of OTC vaginal tablets containing miconazole or clotrimazole was also associated with a nominally shorter AGD, albeit not statistically significant, and shortest in boys that had been exposed during the MPW compared to outside this developmental window (Mogensen et al., 2017).

It should be noted that the aforementioned study, as well as other relevant epidemiological studies of azole fungicides and potential reproductive effects in humans, has relied on a small number of cases. As such, our knowledge is still limited when it comes to associations between developmental azole exposure and adverse reproductive outcomes in humans, including hypospadias or cryptorchidism at birth, or reduced fertility or testis cancers later in life. But several epidemiological studies, again with small case numbers, have found no overall increased risk of hypospadias or cryptorchidism following azole treatment during pregnancy (Mastroiacovo et al., 1996; Czeizel et al., 2004; Carter et al., 2008; Nørgaard et al., 2008; Howley et al., 2016; Liu et al., 2020). Based on these seemingly disparate studies of low statistical power, larger prospective studies investigating the potential adverse reproductive effects of azole medications are needed to be able to form any solid conclusions.

**FIGURE 1:**
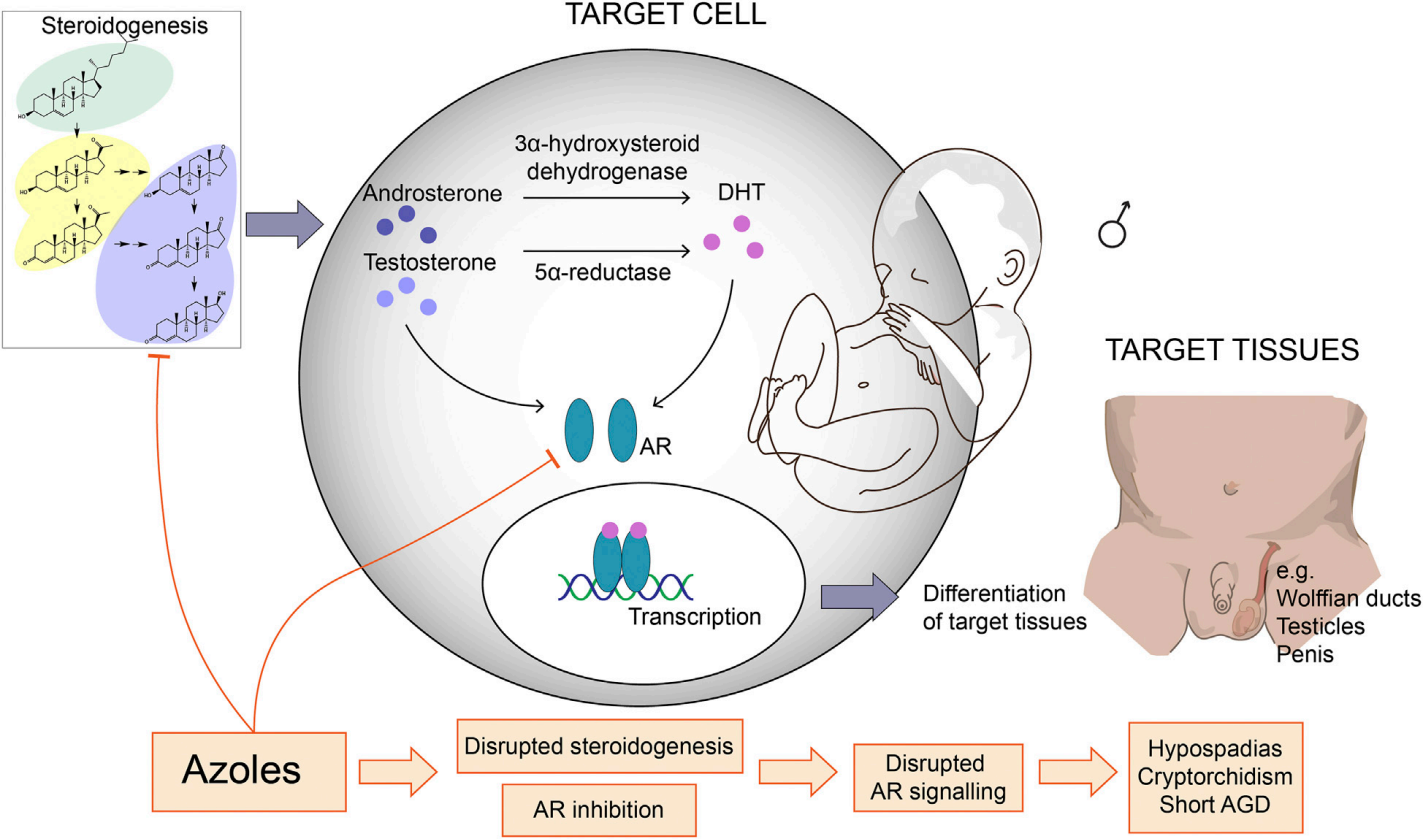
Azole fungicides with anti-androgenic effects and their potential mechanisms of action. Many azole fungicides can inhibit CYP enzyme activity and thereby disrupt steroidogenesis and, ultimately, steroid hormone profiles. This may lead to reduced androgen synthesis and signaling in androgen-sensitive target cells. Certain azoles can also antagonize the androgen receptor (AR) thereby interfere with AR activation and gene regulation in target cells. Since androgen signaling is crucial for masculinization during development, these molecular effects can impede on male development leading to reproductive disorders. AGD = anogenital distance, DHT = dihydrotestosterone.

Numerous studies using rats or mice have investigated the endocrine disrupting potentials of azole fungicides from both agriculture and medicine, and the observed adverse reproductive outcomes are many and diverse (Figure 1). Several azoles induce anti-androgenic effects such as short male AGD following developmental exposure (Vinggaard et al., 2005; Laier et al., 2006; Taxvig et al., 2007; Taxvig et al., 2008; Hass et al., 2012; Johansson et al., 2021a), but surprisingly also longer AGDs in both male and female offspring with certain azole fungicides (Noriega et al., 2005; Laier et al., 2006; Goetz et al., 2007; Taxvig et al., 2007; Christiansen et al., 2009; Hass et al., 2012; Melching-Kollmuss et al., 2017). Developmental exposure to azoles have also been shown to cause nipple retention in male rat offspring (Noriega et al., 2005; Vinggaard et al., 2005; Laier et al., 2006; Taxvig et al., 2007; Hass et al., 2012), which is a biomarker for compromised androgen action (Schwartz et al., 2021). Azoles can also cause increased incidents of malformed external genitalia and hypospadias (Noriega et al., 2005; Laier et al., 2006), as well as delayed pubertal onset (Melching-Kollmuss et al., 2017; Johansson et al., 2021a), and fewer motile sperm (Taxvig et al., 2007; Melching-Kollmuss et al., 2017).

Another frequently observed effect in rats is changes to steroid hormone levels in both serum and reproductive organs, specifically the testes. Common observations include decreased testosterone levels and increased progesterone (Vinggaard et al., 2005; Vinggaard et al., 2006; Laier et al., 2006; Taxvig et al., 2007; Taxvig et al., 2008; Draskau et al., 2021), but also decreased estrogen in plasma (Taxvig et al., 2008; Draskau et al., 2019; Draskau et al., 2021). These *in vivo* effects fit with what we know about the affinity towards CYP enzymes for many azoles, as revealed by numerous *in vitro* studies (Loose et al., 1983; Mason et al., 1985; Mason et al., 1987; Ayub and Levell, 1989a; Kragie et al., 2002; Zarn et al., 2003; Trösken et al., 2006; Kjærstad et al., 2010; Ohlsson et al., 2010; Van der Pas et al., 2012; Gal and Orly, 2014; Munkboel et al., 2019). This also extends to human fetal testis explant studies with ketoconazole, imazalil, prochloraz, and propiconazole, where exposure significantly lowered testosterone production (up to 90%) compared to vehicle controls (Mazaud-Guittot et al., 2013; Gaudriault et al., 2017). As for elevated progesterone, this is of potential importance for two reasons. Firstly, progesterone does not only act as a ligand for the progesterone receptor (PR), but has also been shown to have off-target effects on other nuclear receptors and give rise to various effects *in vivo*, including masculinization effects in female offspring arising from exposure to the progestin medroxyprogesterone (Wilkins et al., 1958; Money and Mathews, 1982; Kuhl, 2005). Secondly, because elevated progesterone may also lead to elevated androgen levels (Auchus and Chang, 2010), which help explain masculinization effects in female rat offspring exposed to prochloraz (Laier et al., 2006; Melching-Kollmuss et al., 2017). Finally, disrupted progesterone levels is also of importance in that it is intimately involved in regulating parturition. In fact, many azoles are known to disrupt parturition or induce dystocia in rats, likely by maintained high progesterone levels when a significant drop is required to induce parturition (Wolf et al., 1999; Noriega et al., 2005; Taxvig et al., 2007; Melching-Kollmuss et al., 2017; Johansson et al., 2021a).

Certain azoles are potent AR antagonists *in vitro* (Kjærstad et al., 2010; Roelofs et al., 2014; Draskau et al., 2019). Many of these are agricultural azoles, but they also include some pharmaceuticals, albeit they appear to be less potent AR antagonists. For instance, itraconazole (Williams et al., 2017; U.S. Environmental Protection Agency, 2021) and ketoconazole (Eil, 1992) (with some opposing evidence (Ayub and Levell, 1989b; Kjærstad et al., 2010)) seem able to interfere with the AR, whereas clotrimazole, miconazole, and fluconazole do not (Ayub and Levell, 1989b; Eil, 1992; Kjærstad et al., 2010; Roelofs et al., 2014). This is important since, as discussed above, fetal male reproductive development in humans is less dependent on testosterone as DHT can be synthesized from the intermediate androsterone coming from the placenta, adrenal, or liver. Notably, however, steroidogenesis disruptors can also be envisioned to perturb synthesis in other tissues and organs, as well as other biosynthesis pathways, including the backdoor pathway. Nevertheless, humans may not be as susceptible to disruption by chemicals perturbing testosterone synthesis by the testes as mice and rats are. The AR, on the other hand, is downstream of DHT action and consequently chemicals blocking DHT-dependent activation of the AR can have detrimental effects in the developing fetus irrespective of androgen synthesis. Thus, despite significant differences in androgen synthesis between mice/rats and humans, potential direct effects on nuclear receptors such as the AR raise further concern about the human health-related effects of exposure to azole fungicides.

*In vitro* and *in vivo* studies clearly demonstrate the endocrine disrupting potential of azole fungicides. Then, despite the fact that many azole fungicides are not readily absorbed (for instance from vaginal applicators) and are often rapidly metabolized, concerns still remain regarding indiscriminate use of azole fungicides, especially during pregnancy. For instance, an OTC azole fungicide such as clotrimazole has shown strong endocrine disrupting effects *in vitro* and *in vivo* at very low concentrations, even below human therapeutically used concentrations (Munkboel et al., 2019; Draskau et al., 2021). A second example, fluconazole, also displays endocrine disrupting potential (Mogensen et al., 2017; Munkboel et al., 2019), almost completely avoids metabolization (Bury et al., 2021), and can rapidly penetrate from plasma into female genital organs including uterus, ovary, and oviduct (Mikamo et al., 1999). These examples are cause for some concern regarding azole use during pregnancy and concomitant exposure of the unborn child.

## Non-Classical Mechanisms of Azole Fungicides

When assessing the endocrine disrupting potential of chemicals it is common to primarily consider the classic estrogenic, androgenic, thyroid, and steroidogenic modalities. This does not exclude that other modalities could be relevant for endocrine disruption, however, and many other signaling pathways have been highlighted as important to consider in this context (Martyniuk et al., 2022). Our knowledge remains limited when considering the extent to which other mechanisms relevant for endocrine disruption can cause adverse health effects, but with respect to azole fungicides several alternative signaling pathways may be relevant.

Evidence exists that many azoles are capable of disrupting important evolutionary conserved pathways. For instance, the antifungal drug itraconazole can interfere with Hedgehog (HH) signaling (Tiboni et al., 2006; Johansson et al., 2021b), whereas ketoconazole can, for instance, affect sex-specific retinoic acid (RA) signaling in developing mouse testes (Bowles et al., 2006; Koubova et al., 2006). However, interference with these important morphogenic pathways also raises the question of teratogenicity.

Potent HH and RA disruptors, including specific azoles, are known to induce malformations and severe developmental toxicity in both animal models (Menegola et al., 2006; Tiboni et al., 2006, Tiboni et al., 2008; Dimopoulou et al., 2017) and humans (Mastroiacovo et al., 1996; Kazy et al., 2005; Carter et al., 2008; Nørgaard et al., 2008; Giavini and Menegola, 2010; Mølgaard-Nielsen et al., 2013; Howley et al., 2016). As such, chemicals acting by these modes of action would likely cause teratogenic effects at doses below or equal to those that would disrupt reproductive development. Still, considering the fact that human exposure often consists of a larger mixture of chemicals each at low concentrations, likely below concentrations that will induce teratogenicity, it can be speculated that several azoles or other chemicals interfering with these signaling pathways simultaneously can actually disrupt reproductive development. On the other hand, the importance of these modalities for medicinal drugs, which are often used at high concentrations, in inducing reproductive disorders may thus be to a lesser extent.

## Concluding Remarks

Azoles are important agents used to combat fungal infections. Their importance for these applications should not be underestimated, not least considering that fungal infections can be very difficult to control or overcome. This short review is not intended to question the value of azole-containing drugs in treating these conditions. Rather, it is intended to highlight the fact that azole compounds can be very potent endocrine disruptors and act on several different signaling pathways that are crucial for normal development, herein with focus on reproductive development. This review has also emphasized a potential concern about OTC azole fungicides in particular, since indiscriminate use during pregnancy could impact reproductive development; perhaps not in isolation, but could lead to problematic outcomes if used in combination with other endocrine disrupting compounds such as analgesics and general exposure to environmental chemicals. Perhaps we would be better served by applying a cautionary principle.
